# Beyond the reference: gene expression variation and transcriptional response to RNA interference in *Caenorhabditis elegans*

**DOI:** 10.1093/g3journal/jkad112

**Published:** 2023-05-23

**Authors:** Avery Davis Bell, Han Ting Chou, Francisco Valencia, Annalise B Paaby

**Affiliations:** School of Biological Sciences, Georgia Institute of Technology, 950 Atlantic Dr NW, EBB Building, Atlanta, GA 30332, USA; School of Biological Sciences, Georgia Institute of Technology, 950 Atlantic Dr NW, EBB Building, Atlanta, GA 30332, USA; School of Biological Sciences, Georgia Institute of Technology, 950 Atlantic Dr NW, EBB Building, Atlanta, GA 30332, USA; School of Biological Sciences, Georgia Institute of Technology, 950 Atlantic Dr NW, EBB Building, Atlanta, GA 30332, USA

**Keywords:** *Caenorhabditis elegans*, RNA-seq, natural genetic diversity, expression variation, RNA interference

## Abstract

Though natural systems harbor genetic and phenotypic variation, research in model organisms is often restricted to a reference strain. Focusing on a reference strain yields a great depth of knowledge but potentially at the cost of breadth of understanding. Furthermore, tools developed in the reference context may introduce bias when applied to other strains, posing challenges to defining the scope of variation within model systems. Here, we evaluate how genetic differences among 5 wild *Caenorhabditis elegans* strains affect gene expression and its quantification, in general and after induction of the RNA interference (RNAi) response. Across strains, 34% of genes were differentially expressed in the control condition, including 411 genes that were not expressed at all in at least 1 strain; 49 of these were unexpressed in reference strain N2. Reference genome mapping bias caused limited concern: despite hyperdiverse hotspots throughout the genome, 92% of variably expressed genes were robust to mapping issues. The transcriptional response to RNAi was highly strain- and target-gene-specific and did not correlate with RNAi efficiency, as the 2 RNAi-insensitive strains showed more differentially expressed genes following RNAi treatment than the RNAi-sensitive reference strain. We conclude that gene expression, generally and in response to RNAi, differs across *C. elegans* strains such that the choice of strain may meaningfully influence scientific inferences. Finally, we introduce a resource for querying gene expression variation in this dataset at https://wildworm.biosci.gatech.edu/rnai/.

## Teaser

This study evaluates how genetic differences among five wild strains affect gene expression in *C. elegans*, in general and after inducing the RNA interference (RNAi) response. This investigation revealed substantial differences in gene expression across strains and a complex and strain specific transcriptional response to RNAi. Gene expression in *C. elegans* varies, generally and in response to RNAi, such that choice of strain may meaningfully influence scientific conclusions. Bell et al., introduce a resource for exploring this dataset at https://wildworm.biosci.gatech.edu/rnai/.

## Introduction

Research in the model organism *Caenorhabditis elegans* has yielded insight into myriad aspects of biology, particularly development, genetics, and molecular biology (Corsi *et al*. 2015). Historically, much of this work has been conducted in a single isogenic strain, the laboratory strain N2 (Barriere and Felix 2005b; Andersen *et al*. 2012). However, *C. elegans* harbors significant intraspecific genetic diversity (Barriere and Felix 2005a, 2005b; Andersen *et al*. 2012; Crombie *et al*. 2019; Lee *et al*. 2021), and in the last decade, *C. elegans* has also been established as a powerful system for elucidating connections between genotypes and phenotypes (Barriere and Felix 2005a, 2005b; Gaertner and Phillips 2010; Andersen *et al*. 2012; Cook *et al*. 2017; Crombie *et al*. 2019; Evans *et al*. 2021a; Lee *et al*. 2021; Andersen and Rockman 2022). Natural genetic variation exists for practically any organismal trait measurable in *C. elegans* (Andersen and Rockman 2022), for example, responsiveness to toxins, metals, drugs, and other stressors (Zdraljevic *et al*. 2017; Hahnel *et al*. 2018; Webster *et al*. 2019; Zdraljevic *et al*. 2019; Evans and Andersen 2020; Na *et al*. 2020; Dilks *et al*. 2021; Evans *et al*. 2021b); behavior (McGrath *et al*. 2009; Bendesky *et al*. 2012; Ghosh *et al*. 2015); transgenerational mortality traits (Frezal *et al*. 2018; Saber *et al*. 2022); and efficiency in RNA interference (RNAi) (Tijsterman *et al*. 2002; Felix 2008; Elvin *et al*. 2011; Felix *et al*. 2011; Paaby *et al*. 2015).

Naturally, molecular phenotypes that act as intermediaries between genotypes and organismal traits, such as gene expression, also vary across strains. Studies from recombinant inbred lines (Rockman *et al*. 2010; Vinuela *et al*. 2010; Evans and Andersen 2020) and, more recently, RNA sequencing of 207 wild strains (Zhang *et al*. 2022) have identified numerous expression quantitative trait loci (eQTL) that encode differences in gene expression. How such expression differences manifest across different strains, whether they offer clues into functional differentiation, and how genetic differences compare to environmentally induced differences in gene expression or mediate gene expression responses to environmental stimuli remain interesting questions. These questions require genome-wide characterization of gene expression in multiple strains under multiple conditions.

One phenomenon of particular interest is RNAi, a mechanism of gene expression regulation triggered by environmental or endogenous sources of double-stranded RNA with broad-reaching influence over diverse aspects of organismal biology (Wilson and Doudna 2013; Billi *et al*. 2014). RNAi was discovered in *C. elegans* (Fire *et al*. 1998), but competency in response to environmental triggers is highly variable across wild *C. elegans* strains (Tijsterman *et al*. 2002; Felix 2008; Elvin *et al*. 2011; Felix *et al*. 2011; Paaby *et al*. 2015). Previous work showed that a loss-of-function mutation in Argonaute RNAi effector gene *ppw-1* is largely responsible for the near-complete failure of Hawaiian strain CB4856 to mount an RNAi response against germline targets (Tijsterman *et al*. 2002), and later work characterized the failure in CB4856 as a much delayed, rather than absent, response (Chou *et al*. 2022). Other strains incompetent for germline RNAi exhibit distinct modes of RNAi failure with distinct genetic bases (Elvin *et al*. 2011; Pollard and Rockman 2013; Chou *et al*. 2022). Even as wild strains vary in overall competency for germline RNAi, strain-to-strain differences in RNAi phenotypic penetrance are also highly dependent on the target gene; whether these differences arise from strain-specific developmental consequences of gene knockdown or strain-specific differences in target-dependent RNAi efficacy is unclear (Paaby *et al*. 2015). How this phenotypic variation in RNAi response is reflected in genome-wide transcriptional changes upon RNAi induction remains a largely open question.

Here, we evaluate how the genotype (strain) and induction of the RNAi response affect the *C. elegans* transcriptome. We also consider how reliance on the reference genome, derived from the laboratory strain N2, might constrain estimates of gene expression in wild strains and how a focus on N2 in studies of RNAi might limit inferences about RNAi biology within *C. elegans* generally. To investigate these questions and to provide a public resource for interrogating transcriptional variation in this system, we performed RNA sequencing on 5 *C. elegans* strains with varying competency in germline RNAi, both in the control condition and under RNAi treatment targeting 2 germline-expressed genes.

## Materials and methods

### Sample preparation and sequencing

#### Worm strains and husbandry

Strains used in this study include wild strains CB4856, EG4348, JU1088, and QX1211 (gifts from Matthew Rockman) and wild-type laboratory strain N2 (gift from Patrick McGrath). Prior to beginning the RNA-seq relevant experiments, worms were cultured under standard conditions (Stiernagle 2006) except that plates used for non-N2 wild strains were made with 1.25% agarose to prevent burrowing. All strains except for QX1211 were maintained at 20°C; QX1211 was maintained at 18°C to prevent induction of its mortal germline phenotype (Frezal *et al*. 2018). Worms were cultured for at least 3 generations without starvation before RNAi induction and RNA sequencing. Following culture expansion, all strains were handled under identical conditions for RNAi induction and sample collection (see below).

#### RNAi

RNAi was induced via feeding and was carried out on plates at 20°C following established methods (Kamath *et al*. 2001; Ahringer 2006). Worms were fed HT115 *Escherichia coli* bacteria that had been transformed with the empty pL4440 vector or the pL4440-derived vectors *par-1* (H39E23.1) and *pos-1* (F52E1.1) from the Ahringer feeding library (Kamath and Ahringer 2003). Bacterial cultures were prepared by streaking from frozen stocks onto LB agar with carbenicillin (25 µg/mL) and tetracycline (12.5 mg/mL); next, 5–10 colonies from <1-week-old plates were used to inoculate liquid cultures of LB broth with carbenicillin (50 µg/mL) and tetracycline (12.5 mg/mL), which were then incubated with shaking at 37°C for 16–18 h and finally amplified with carbenicillin (50 µg/mL) for 6 h at a 1:200 dilution. Ten-centimeter agar feeding plates with 1 mM IPTG (Ahringer 2006) were seeded with the RNAi bacterial cultures, then used within 44–78 h after incubation in the dark. Worm strains reared under standard conditions were bleached on day 1 to synchronize, then bleached again on day 4 (Stiernagle 2006). On day 5, L1s were transferred to the RNAi plates. All strains were exposed to RNAi in this way at the same time. For library preparation, 6 plates per strain and treatment combination were divided into 3 biological replicates, with 2 plates per replicate.

#### RNA library preparation and sequencing

As previously described (Chou *et al*. 2022), synchronized hermaphrodites reared on RNAi feeding plates were washed off at the first sign of egg laying, washed twice with M9 buffer, and stored in TRIzol (Invitrogen #15596026) at −80°C until RNA extraction. Age synchronization was conducted similarly to other studies of transcription across *C. elegans* strains (Zhang *et al*. 2022) via close monitoring of culture plates to identify the point at which most animals were gravid and the earliest embryos were laid. RNA was extracted from all samples at the same time using TRIzol (Invitrogen #15596026) and RNeasy columns (Qiagen #74104) (He 2011). cDNA and sequencing libraries were generated from 500 ng of fresh RNA samples with 10 cycles of PCR with the NEBNext Ultra II Directional RNA Library Prep Kit for Illumina (NEB #7760). After quality checking using an Agilent 2100 Bioanalyzer, library fragments were size-selected via BluePippin (Sage Science). Single-end 75-bp reads were sequenced on an Illumina NextSeq at the Molecular Evolution Core facility at the Georgia Institute of Technology.

### Analysis

#### Analytical approach

We considered multiple state-of-the-art pipelines to align RNA-seq data and quantify expression. Because the 4 wild strains in our study are diverged from the N2 reference genome by differing degrees (Cook *et al*. 2017), we required a method that could evaluate N2 data and non-N2 data over a range of variation without bias. One variant-aware option for quantifying RNA expression is to consider only RNA-seq reads that align to exactly 1 position on the reference genome (unique mappers) using STAR (Dobin *et al*. 2012) and to discard reads not uniquely aligning to the same position after nonreference variants are swapped into the read using WASP (van de Geijn *et al*. 2015). We explored this approach with our data. Specifically, we used STAR v2.7.5a with nondefault parameters –outFilterMismatchNmax 33 –seedSearchStartLmax 33 –alignSJoverhangMin 8 –outFilterScoreMinOverLread 0.3 –alignIntronMin 40 –alignIntronMax 2200 –waspOutputMode SAMtag –varVCFfile (VCF containing SNPs from all 4 nonreference strains); these latter parameters implemented WASP from within STAR.

A second option is to generate strain-specific transcriptomes that incorporate known variants from each strain into the reference genome and use those to quantify transcript expression via pseudoalignment; this approach permits reads to map to multiple locations (Bray *et al*. 2016; Patro *et al*. 2017). We do not compare the STAR–WASP approach to this pseudoalignment approach here; high-level results were similar between the approaches. For our final analysis, we chose the second option for multiple reasons: (1) pseudoalignment approaches are at least as accurate at estimating expression while being computationally more efficient (Bray *et al*. 2016; Patro *et al*. 2017); (2) pseudoalignment approaches take into account the large fraction of reads that align to multiple loci in the genome (Bray *et al*. 2016; Patro *et al*. 2017); and (3) our specific generation of strain-specific transcriptomes enabled us to include insertion/deletion polymorphisms (INDELs), whereas WASP ignores INDELs (van de Geijn *et al*. 2015). Including INDELs was particularly relevant in this study, as 8,195–67,267 INDELs differentiate the 4 nonreference strains from the reference genome [*C. elegans* Natural Diversity Resource (CeNDR) 20210121 release] (Cook *et al*. 2017).

The following methods detail generation of strain-specific transcriptomes and pseudoalignment to quantify expression at individual genes. A subset of these methods and data overlap with our recent RNAi-focused study, which examined expression variation at specific RNAi genes (Chou *et al*. 2022).

#### Strain-specific transcriptomes

As previously described (Chou *et al*. 2022), we used SNPs and INDELs from CeNDR (20210121 release) (Cook *et al*. 2017) to update the N2 reference genome (ws276 release) (Harris *et al*. 2020) to generate strain-specific transcriptomes using the software g2gtools (v0.1.31 via Conda v4.7.12, Python v2.7.16) (https://github.com/churchill-lab/g2gtools). Specifically, INDELs were added to the reference genome with *g2gtools vcf2chain* and SNPs with *g2gtools patch*. INDELs were added to the SNP-updated genome with *g2gtools transform*. We generated strain-specific GTFs from the strain-specific FASTAs with *g2gtools convert* and generated strain-specific transcriptomes from these GTFs with gffread (v0.12.7) (Pertea and Pertea 2020).

The Nextflow workflow performing this process is available in this project's code repository (https://github.com/averydavisbell/wormstrainrnaiexpr) at *workflows/strainspectranscriptome*.

#### Gene expression quantification

Transcript-level quantification, used downstream for gene-level estimates, was performed using Salmon (v1.4.0) (Patro *et al*. 2017), as we previously detailed (Chou *et al*. 2022). First, we trimmed Illumina TruSeq adapters from RNA-seq reads with Trimmomatic (v0.3.9) (Bolger *et al*. 2014), parameters *ILLUMINACLIP:TruSeq3- SE.fa:1:30:1.* Strain-specific transcriptomes were used to generate Salmon index files with command *salmon index* with options *–k 31 –keepDuplicates* (all others default; no decoy was used). Salmon transcript quantification *salmon quant* was performed with options *–l SR –dumpEq*, *–rangeFactorizationBins 4*, *–seqBias*, and *–gcBias* and library-specific fragment length arguments *–fldMean* and *–fldSD*.

The Nextflow workflow generating strain-specific transcriptomes also generates strain-specific Salmon indexes; the Nextflow workflow performing transcript quantification is available in this project's code repository at *workflows/strainspecsalmon*.

#### Differential expression analysis

Differential expression analyses were performed in R (v4.1.0) (R Core Team 2021) using the DESeq2 package (v1.32.0) (Love *et al*. 2014). We imported transcript quantification data into DESeq2 using the tximport package (v1.20.0) (Soneson *et al*. 2015), which adds Salmon-specific transcript length normalizations to DESeq2's sample-wise RNA quantification normalization and converts Salmon's transcriptome quantification estimates to gene-level quantification estimates. Genes with fewer than 10 estimated reads across all samples (summed) were excluded from downstream analyses, retaining 18,589 genes. Principal components analysis (PCA) was performed using the top 500 most variably expressed genes across all samples after DESeq2's variance-stabilizing transformation (*vst* function), which was performed blind to the experimental design.

We used DESeq2's likelihood ratio tests to determine whether genes were differentially expressed based on strain in the control condition and whether the interaction of strain and treatment was significant. For strain-wise significance, control sample counts were modeled with the negative binomial model


$$\mathrm{lo}g_{2}(q_{ij})=\beta_{i}x_{j}+1,$$

which was compared to the reduced (null) model


$$\mathrm{lo}g_{2}(q_{ij})=1.$$

Here, for gene *i*, sample *j*, *q* is proportional to the actual concentration of RNA fragments for a gene (derived by DESeq2 from input counts and error modeling) (Love *et al*. 2014). *β_i_* gives the log_2_ fold changes for gene *i* corresponding to strain *x.* A total of 15,654 genes were sufficiently detected in the control samples to be included in this analysis (the remainder were excluded by DESeq2's *P*-value correcting methods).

To evaluate strain–treatment interactions, all sample counts were modeled with the negative binomial model


$$\mathrm{lo}g_{2}(q_{ij})\;=\beta_{1i}x_{j}+\beta_{2i}y_{j}+\beta_{3i}x_{j}y_{j},$$

which was compared to the reduced model


$$\mathrm{lo}g_{2}(q_{ij})=\beta_{1i}x_{j}+\beta_{2i}y_{j}.$$

Here, the symbols are as in the first set of equations, with the additions that *y* corresponds to the RNAi treatment; *xy* to the strain–treatment interaction; and *β*_1_ to the strain effect, *β*_2_ to the treatment effect, and *β*_3_ to the interaction effect.

In both likelihood ratio tests, genome-wide adjusted *P*-values were determined by DESeq2's multiple testing correction. Genes were considered differentially expressed if the *P*-value was less than 0.1.

On the same datasets, we assessed differential expression within strains using DESeq2's Wald's tests of contrasts between treated (*par-1* or *pos-1* RNAi) and control (empty vector) samples. Genes were considered significantly differentially expressed if, after log_2_ fold change shrinkage using the “ashr” method from the package ashr (v2.2-47) (Stephens 2017), their absolute value fold change was greater than 1.5 and genome-wide adjusted *P*-value (FDR) was less than 0.1.

The script performing these analyses is available in this project's code repository at *diffexp_lrt_straintreat_salmon_deseq2.R*.

#### Transcriptional age estimates

We used R package RAPToR (v1.2.0) (Bulteau and Francesconi 2022) to estimate the age of each sample based on its gene expression profile by comparing DESeq2 *vst*-normalized total count data to the *C. elegans* young adult age benchmarks provided with the package; age estimates were robust to several transformations of the count data. Specifically, we used function *ae* with age ruler Cel_YA_2 from RAPToR companion package wormRef (v0.5.0).

#### DNA sequence coverage estimation and identification of low-coverage and missing genes

We examined DNA sequence coverage within genes in CeNDR (Cook *et al*. 2017) BAM files (20210121 release); these files correspond to the same strains as in our study except in the case of EG4348, where CeNDR sequenced genetically identical strain EG4349. We note, of course, that CeNDR DNA alignments were made directly to the N2 genome; we used the variants discovered therein to build our genotype-specific pseudotranscriptomes. To get per-gene DNA sequence coverage, we first generated a file containing the nonoverlapping, nonduplicated locations of all genes’ RNA generating sequences by determining the locations of all merged exons genome-wide using GTFtools (v0.8.5) (Li 2018) (http://www.genemine.org/gtftools.php). Then, we determined the mean per-base coverage of each of these regions using Mosdepth v0.3.2 (Pedersen and Quinlan 2018) with default options with the exception of setting *–flag 1540*, which excludes unmapped reads, PCR duplicates, and QC failures. Finally, we computed the per-gene coverage as


$$\frac{\Sigma (\mathrm{coverage}\;\mathrm{per}\;\mathrm{merged}\;\mathrm{exon}*\mathrm{length}\;\mathrm{of}\;\mathrm{merged}\;\mathrm{exon})}{\Sigma \;\mathrm{length}\;\mathrm{merged}\;\mathrm{exons}\;\mathrm{in}\;\mathrm{gene}}.$$

To delineate a set of low-DNA-coverage genes, we median-normalized the coverage within strains and flagged any with <25% of the median coverage (i.e. median-normalized coverage < 0.25) as low coverage. Genes were classified as putatively missing from nonreference strain genomes if they had raw coverage estimates of exactly 0.

The workflow running this analysis is available in this project's code repository at *workflows/mosdepthmergedexons*; this workflow performs custom gene-level analysis steps by calling an R script available in this project's code repository at *exploregenecoverage_fromexons.R*. The scripts determining overlap with differentially expressed genes and 0-coverage genes are available in this project's code repository at *de_dnacov_overlap.R* and *exploregencoverage_fromexons_lowend.R*.

#### “Off” gene analysis

To identify genes putatively unexpressed in 1 or more strains despite being expressed in others (“off” genes), we first identified all genes differentially expressed between any 2 strains in the control condition (Wald's test comparing each strain pair, genome-wide adjusted *P* < 0.1). The rationale was that genes significant for differential expression between strain pairs must have meaningful expression in at least 1 strain; we employed this standard to avoid inclusion of genes that are simply not expressed or expressed at a very low level regardless of strain. We then determined the average variance-stabilizing transformed (DESeq2 function *vst*) expression across all samples from all 3 treatments within each strain for these genes and identified those with 0 mean expression. (These genes, of course, also have 0 estimated expression prior to *vst* normalization.) Genes with strain-wise differential expression and 0 expression within a strain comprise the “off” gene set. (This process identified an additional 6 genes that fell just short of significance in the global analysis for differential expression in the likelihood ratio test described above.) We then interrogated these genes for overlap with low DNA coverage and differential expression under RNAi treatment.

The script performing these analyses is available in this project's code repository at *offgenes_straintreatDE_deseq2_dnacov.R*.

#### Gene set enrichment analysis

We performed gene set enrichment analysis of genes differentially expressed upon RNAi treatment using WormBase's enrichment analysis tool (Angeles-Albores *et al*. 2016; Harris *et al*. 2020) (https://wormbase.org/tools/enrichment/tea/tea.cgi). We analyzed genes upregulated and downregulated on each RNAi treatment in all 5 strains (20 analyses total; 5 strains × 2 treatments × 2 directions of differential expression). Upregulated genes were those with higher expression on a treatment, with a fold change > 1.5 vs control and adjusted *P*-value < 0.1; downregulated genes were those with lower expression on a treatment, with a fold change < −1.5 vs control and adjusted *P*-value < 0.1 (see *Differential expression analysis*). The background gene set for all analyses was the 18,529 genes included in overall differential expression analyses. All gene set enrichment–related outputs were saved, and the enrichment results table’s (‘Download results table here’) outputs were combined across strains for visualization.

The script performing this limited downstream processing is available in this project's code repository at *exploreGeneSetEnrichmentResults.R*.

#### High-performance computation

Computationally intensive analyses were performed on the infrastructure of PACE (Partnership for an Advanced Computing Environment), the high-performance computing platform at the Georgia Institute of Technology. These analyses comprised pseudotranscriptome generation, expression quantification, DNA sequence coverage estimation, and their related computational tasks.

#### Figures and website

Figures were made in R (v4.1.0) (R Core Team 2021) using packages ggplot2 (v3.3.6) (Wickham 2016), data.table (v1.14.3) (Dowle and Srinivasan 2022) (https://r-datatable.com), DESeq2 (v1.32.0) (Love *et al*. 2014), cowplot (v1.1.1) (Wilke 2020), ggVennDiagram (v1.2.0) (Gao 2021), eulerr (v6.1.1) (Larsson 2021), and ggpattern (v1.0.1) (FC *et al*. 2022), with color schemes developed using RColorBrewer (v1.1-3) (Neuwirth 2022) and Paul Tol's color palettes (https://personal.sron.nl/∼pault/). The interactive website that enables exploration of the data from this study was developed using Shiny (Chang *et al*. 2022).

## Results and discussion

To investigate natural variation in both gene expression and response to exogenous RNAi, we performed RNA sequencing on 5 isogenic *C. elegans* strains in 3 conditions: RNAi targeting the germline genes *par-1* and *pos-1* and the untreated condition. We included the RNAi-competent reference strain N2 and 4 wild strains with varying competency to germline RNAi (Paaby *et al.* 2015; Chou *et al.* 2022): JU1088 (highly competent), EG4348 (moderately competent), and CB4856 and QX1211 (largely incompetent). These wild strains also vary in divergence from N2, representing some of the least (JU1088) and most (QX1211) divergent strains (variants per kilobase vs N2 genome: 0.82, 1.40, 1.99, and 4.20, respectively, from CeNDR data (Cook *et al*. 2017)). To minimize potentially confounding effects of different developmental timing among strains, we stage-matched all samples to the first sign of egg laying, then verified developmental consistency by estimating sample age from the gene expression profiles (Bulteau and Francesconi 2022) (Supplementary Fig. 1). To limit bias arising from differences between non-N2 sequencing reads and the N2 reference genome in our analysis, we first created strain-specific transcriptomes by inserting known single nucleotide and insertion/deletion variants from CeNDR (Cook *et al*. 2017) into the reference genome. Then, we pseudoaligned the RNA reads to these strain-specific transcriptomes to quantify per-gene RNA expression in each strain in each condition and estimated differential expression based on strain, RNAi treatment, and their interaction.

### Genotype (strain)-wise expression variation predominates and identifies potentially functionally diverged genes

**Fig. 1.**
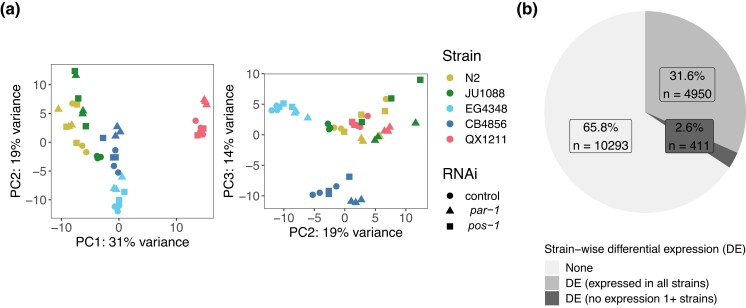
Genotype (strain) dominates expression variation across 5 *C. elegans* strains treated with RNAi targeting the genes *par-1* and *pos-1* or the empty vector control (*n* = 3 biological replicates in each condition in each strain). a) PCA of gene expression. PCs 1 vs 2 (left) and 2 vs 3 (right) of PCA of the 500 most variably expressed genes are plotted; the proportion of variance explained is noted on the axes. b) In the control condition, 34.2% of 15,654 nominally expressed genes are differentially expressed across strains (genome-wide adjusted *P* < 0.1 in a likelihood ratio test between models including and excluding the *strain* term); a subset of these (approximately 2.6% overall) are not expressed at all in at least 1 strain (in any condition, see text for details). Related Supplementary Material: Supplementary File 1 contains the genes differentially expressed based on strain. Supplementary File 2 contains the “off” genes identified as potentially unexpressed in 1 strain but expressed in others.

Overall, genotypic differences between strains explained more gene expression variation than RNAi treatment. We detected nominal expression at 18,589 genes across the full dataset; a PCA of the 500 most variable genes shows distinct strain-wise partitioning of the variation (Fig. 1a). To identify genes with significant expression differences between strains in just the control condition, we compared a model with a term for strain to 1 without (via a likelihood ratio test) for each gene. Of the 15,654 genes included in this control-specific analysis, 5355, or approximately 34%, were differentially expressed across the 5 strains (likelihood ratio test, genome-wide adjusted *P* < 0.1) (Supplementary File 1). This fraction of genes with expression differences between strains is consistent with recent findings that 28% of assayed genes were associated with mappable genetic differences (eQTL) across 207 wild strains (Zhang *et al*. 2022). Other systems, such as flies, also harbor extensive variation in gene expression: a recent study of 200 inbred *Drosophila melanogaster* strains detected strain-wise expression variation in the majority of genes (Everett *et al*. 2020). The experimental and analytical approach matters a great deal; in the *Drosophila* study, many more variable genes were identified using RNA-seq data than microarray data, and only 30–40% of differentially expressed genes were associated with mappable eQTL (Everett *et al*. 2020).

In some cases, the presence vs absence of expression may underpin differential expression across strains; this pattern could indicate strain-wise differences in functional requirements or in developmental timing of expression. We identified such “off” genes as those with 0 mean expression in at least 1 strain (across all conditions) as well as significant strain-wise differential expression between a pair of strains in the control condition (genome-wide adjusted *P* < 0.1). This conservative 0-read threshold reduces the frequency of misclassifying low-expression genes as off; the requirement for differential expression ensures true expression in at least 1 strain. This stringent selection yielded 411 putative “off” genes (Fig. 1b, Supplementary File 2). Most of these genes lacked expression in a single strain: 249 were off in 1 strain, 105 were off in 2 strains, 51 were off in 3 strains, and only 6 genes were expressed in a single strain and off in the others (Supplementary Fig. 2a). We detected 49 genes that were off in N2 but expressed in at least 1 other *C. elegans* strain. The complete functional repertoire of these genes would therefore be invisible in a study using only the N2 strain. Such on/off patterns of gene expression occur in other systems as well; for example, across 144 *Arabidopsis thaliana* strains, thousands of genes showed strong expression in some strains but 0 expression in others (Zan *et al*. 2016).

To assess the potential significance of “off” genes in the context of RNAi response, we investigated whether any genes unexpressed in 1 strain exhibited differential expression within another strain following *par-1* or *pos-1* RNAi treatment. Of the 411 “off” genes, 47 were differentially expressed on RNAi treatment in at least 1 other strain (RNAi differential expression threshold: genome-wide adjusted *P* < 0.1 and fold change > 1.5 for within-strain RNAi treatment vs control comparisons) (Supplementary Fig. 2b). The majority (*n* = 33) of these genes were differentially expressed in only 1 RNAi treatment in 1 strain. However, 1 gene identified by this analysis is W06G6.11 (WBGene00012313), which was “off” in N2 but expressed in the other strains and was significantly upregulated on RNAi against both *par-1* and *pos-1* in RNAi-sensitive strain JU1088 (fold change = 1.9 and genome-wide adjusted *P* = 0.03 for *par-1*; fold change = 3.4 and genome-wide adjusted *P* = 0.003 for *pos-1*). Prior RNA-seq and microarray studies have indicated that W06G6.11 expression may be affected by the activity of Argonaute *alg-1* (Aalto *et al*. 2018), a member of the RNA-induced silencing complex (RISC) involved in endogenous and exogenous short RNA processing (Grishok *et al*. 2001), and also by exposure to pathogens (Engelmann *et al*. 2011; Lee *et al*. 2013). These studies detected W06G6.11 expression in N2, but in samples derived from older adult hermaphrodites relative to the young adults we sampled, a study that included CB4856 also confirmed significantly higher W06G6.11 expression in that strain relative to N2 (Zamanian *et al*. 2018).

This process of identifying genes that are unexpressed in some strains but differentially expressed based on a treatment or phenotype of interest in others might be used to identify candidate genes for other naturally variable phenotypes, perhaps as a complement to genotype-to-phenotype mapping by genome-wide association studies with expression mediation analyses (Evans and Andersen 2020; Zhang *et al*. 2022).

### Reference bias screening increases confidence in differential expression calls

For RNA-seq studies that evaluate wild strains, reliance on a reference strain poses a concern. The main issue is whether the mapping of fewer nonreference strain RNA reads than reference strain reads to a gene arises from true differences in gene expression or from failure of nonreference reads to correctly map to the reference genome due to sequence divergence (reference bias) (Degner *et al*. 2009). Such discrepancies might remain even after the use of genotype-specific transcriptomes. In the case of *C. elegans*, wild strains exhibit a wide range in levels of divergence from the reference strain N2 in the species generally and the strains studied here specifically (Andersen *et al*. 2012; Cook *et al*. 2017; Crombie *et al*. 2019); much of this diversity is located in hyperdivergent haplotypes encompassing 20% of the genome (Lee *et al*. 2021).

**Fig. 2.**
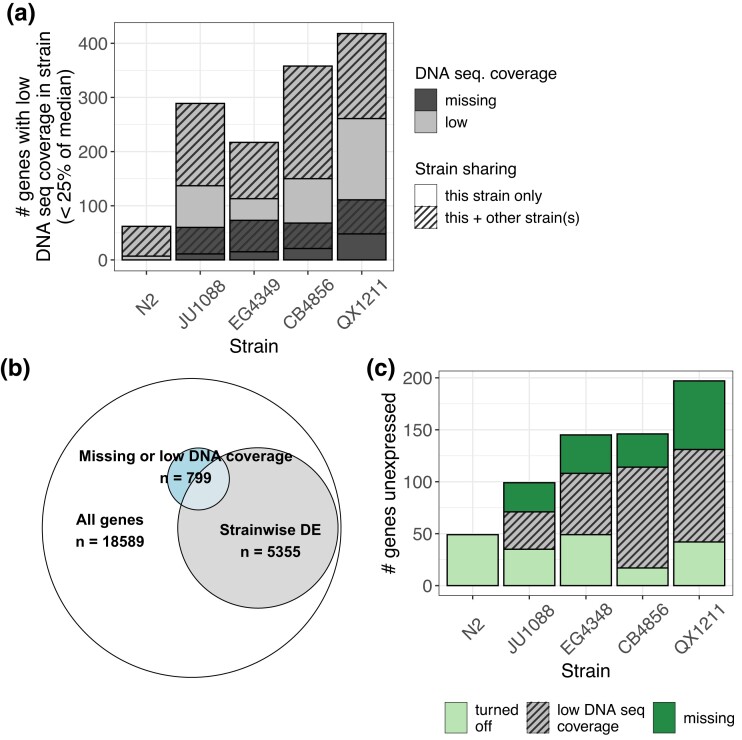
Improving confidence in differential expression calls by integrating DNA alignment data. a) The number of genes with low (<25% of the median) and missing (0 raw coverage) DNA alignment coverage (from CeNDR sequencing (Cook *et al*. 2017)) in each strain of the 18,589 genes included in the expression analysis. Strain note: CeNDR assessed DNA coverage in EG4349, the genetically identical isotype to EG4348. b) The total number of genes differentially expressed based on strain (likelihood ratio test of models including and excluding the *strain* term, genome-wide adjusted *P* < 0.1) and their overlap with genes classified as missing or low DNA coverage in any strain (417 are both differentially expressed across strains and low DNA coverage, hypergeometric enrichment test *P* = 9.8 × 10^−46^). Areas are proportional to the number of observations. c) The number of unexpressed “off” genes per strain, subset into 3 categories: called as turned off at the RNA level with high confidence; missing in the strain genome (0 raw coverage); and called with uncertainty, given low DNA sequence coverage (<25% but >0 median DNA coverage). Related Supplementary Material: Supplementary Fig. 2 shows DNA coverage distributions and cutoffs. Supplementary File 2 contains details on each “off” gene. Supplementary File 3 contains raw per-gene DNA sequence coverage estimates. Supplementary File 4 contains median-normalized per-gene DNA sequence coverage estimates. Supplementary File 5 contains the list of genes flagged as low DNA coverage. Supplementary Files 6 and 7 provide numerical summaries of “off” genes.

To refine our level of confidence in the genes we identified as differentially expressed, we examined our results in the context of alignment quality in the original CeNDR genome sequencing data (Cook *et al*. 2017) (Supplementary Fig. 3, Supplementary Files 3 and 4). For each strain in our study, we curated a list of genes with missing or poor DNA sequence alignment in CeNDR (Cook *et al*. 2017) (Supplementary File 5). Specifically, we classified genes with exactly 0 coverage as missing in that strain's genome; this is a conservative assignment, as even 1 well-aligned DNA sequence read precluded a gene from being classified as missing. We classified genes with more than 0 coverage but less than 25% of the gene-wise median DNA coverage in each strain as low coverage. This process identified a similar set of genes across strains despite the contribution of some strain-to-strain coverage variation (Supplementary Fig. 3, Supplementary File 5). In total, we identified 799 genes as missing or low DNA coverage in 1 or more strains (Fig. 2a).

Were differentially expressed genes associated with poor DNA coverage? Overall, yes: overlap of the missing or low-coverage and strain-wise differentially expressed gene sets revealed significant enrichment (hypergeometric test of enrichment *P* = 9.8 × 10^−46^). However, the absolute number of differentially expressed genes with poor DNA coverage was modest: only 4% of all genes analyzed and 8% of genes with differential expression across strains had missing or low DNA coverage (Fig. 2b). Put another way, 52% of missing or low-DNA-coverage genes were called as differentially expressed, while 29% of all analyzed genes were called as differentially expressed. Further, we note that poor DNA coverage arises from several sources. First, by chance, some genes will be low coverage simply due to stochastic variation in short-read sequencing depth, as reflected in the 62 genes binned as low coverage in N2 mapped to itself (Fig. 2a). Second, sequence divergence between the mapped strain and the reference genome could inhibit alignment (reference bias); this possibility motivates this analysis. Third, the gene could be missing from the strain's genome while present in the N2 reference genome. Not surprisingly, QX1211, the strain most diverged from the N2 reference genome, exhibits the most missing genes and the most low-coverage genes (Fig. 2a, Supplementary File 6).

The set of “off” genes that show 0 expression in some strains may be particularly vulnerable to reference bias, for example, if they were more likely to be pseudogenes in at least 1 strain. In this scenario, poor DNA coverage may be conflated with true expression loss, as accumulated mutations may lead both to poor DNA coverage and consequently poor RNA alignment and to reduced expression through mutation-mediated defunctionalization. Here, when genes are detected as unexpressed, we can make distinctions between (1) missing genes, which we are reasonably confident do not exist in the strain genome; (2) genes for which we may not trust the conclusion of 0 expression because of low DNA coverage and potential bias in RNA read mapping; and (3) true “off” genes, which do not fall into either category and likely represent unbiased expression differences at the RNA level. In this scheme, among the 4 nonreference strains, 17–49 (12–35%) of the originally detected “off” genes are likely truly turned off, 28–66 (22–34%) appear missing from the strain genome, and 36–89 (36–66%) are undetected for an unknown reason but have low DNA coverage and may be influenced by reference bias (Fig. 2c, Supplementary File 7).

As we would expect, all 49 “off” genes in the reference strain N2 were classified as truly unexpressed; none were missing or low coverage (Fig. 2c). Of these, 22 are listed as pseudogenes on WormBase (Harris *et al*. 2020) and may represent alleles that have been pseudogenized in the N2 lineage but remain functional in other strains. One such candidate is Argonaute ZK218.8 (WBGene00013942), which is expressed in strains CB4856 and QX1211 and may reflect functional diversification in RNAi processes across the population (Chou *et al*. 2022). Of the 47 “off” genes with *par-1* or *pos-1* RNAi effects in another strain, a large majority (*n* = 39, 83%) were missing in the genome or were associated with low DNA coverage (Supplementary Fig. 4). This majority represents a slight enrichment relative to the proportion of missing or low-coverage genes within the complete set of “off” genes (286/411 or 70%) (1-sided proportion test with continuity correction: *χ*^2^ = 3.05, df = 1, *P* = 0.04). Enrichment of genome divergence among RNAi-responsive “off” genes supports the hypothesis that genes associated with RNAi are evolving rapidly in *C. elegans* (Chou *et al*. 2022). By adding the missing and low-DNA-coverage filters, we infer that, of genes with an RNAi effect in another strain, 0 (in N2) to 12 (in QX1211) were missing from the strain's genome and 1–6 genes per strain were present but truly unexpressed at the RNA level. These genes might be the most interesting candidates for downstream expression–based studies. This set includes the putative RISC-associated gene W06G6.11 (WBGene00012313) discussed above.

An alternative approach to handling reference bias is to sidestep it by excluding transcripts associated with known hyperdivergent haplotypes (Lee *et al*. 2021; Zhang *et al*. 2022). However, because (1) some genes in hyperdivergent regions had good DNA alignment with low SNP density and others outside the regions had no DNA coverage and (2) our study focuses exclusively on genic regions, we chose a gene-level, strictly coverage-based approach for bias screening. Still, a limitation of our approach (and most others) is that it cannot identify bias associated with elevated RNA levels in diverged or duplicated haplotypes relative to the N2 haplotype. Such bias could occur if reads in nonreference strains come from a gene poorly represented or missing in the reference, which are then spuriously assigned to an incorrect gene with a better match. This type of bias is difficult to define, quantify, and exclude. A powerful alternative approach to making strain-specific pseudotranscriptomes would be to use de novo genome assemblies from the other strains; this approach would permit investigation into genes that are missing from the N2 reference genome, which are necessarily missed by the current approach. Such an assembly is available for CB4856 (Thompson *et al*. 2015; Kim *et al*. 2019) but not yet for all strains. Additionally, as for any arbitrary threshold, our cutoff of <25% median coverage likely produces a mix of false positives and negatives, i.e. genes with low DNA coverage but accurate RNA alignments and genes above the coverage cutoff that are nevertheless skewed by reference bias. While those interested in specific genes would therefore do well to interrogate them further, the DNA coverage approach provides a useful quality control filter for initial analyses of differential expression.

### Complex genotype and target specificity in transcriptional response to RNAi

Wild *C. elegans* strains vary in response to exogenous RNAi. In particular, strains differ widely in competence for RNAi against germline targets delivered by feeding, as measured by phenotypic consequences following putative target knockdown (Tijsterman *et al*. 2002; Felix 2008; Elvin *et al*. 2011; Felix *et al*. 2011; Paaby *et al*. 2015). To assess the transcriptional response to RNAi in worms with variable germline RNAi competencies, we fed worms dsRNA targeting the maternal-effect embryonic genes *par-1* and *pos-1* as well as the empty vector control. Both genes are expressed in the mature hermaphrodite germline and are essential for embryonic viability; in competent animals, RNAi by feeding results in dead embryos (Sijen *et al*. 2001; Paaby *et al*. 2015). Gene expression knockdown of the targets themselves confirmed the previously observed differences in RNAi competency (Paaby *et al*. 2015; Chou *et al*. 2022): under *pos-1* RNAi, *pos-1* expression levels dropped the most in JU1088, followed by N2 and then EG4348; strains CB4856 and QX1211 showed no drop in expression (Supplementary Fig. 5a and 5c). RNAi against *par-1*, which induces a less lethal response (Paaby *et al*. 2015; Chou *et al*. 2022), resulted in a similar though a less strong pattern of *par-1* knockdown (Supplementary Fig. 5b and 5d). These results confirm that strains differ in RNAi response and that the response was target-gene-specific; this target specificity was also evident transcriptome-wide.

**Fig. 3.**
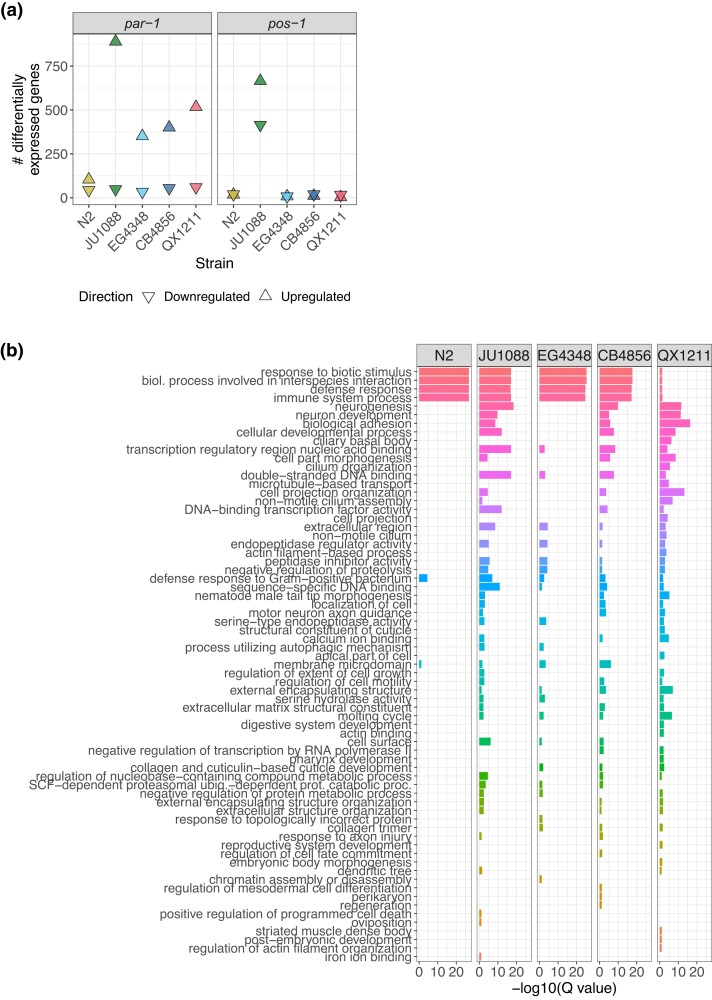
The transcriptional response to dsRNA is highly strain- and target-specific. a) The number of genes up- and downregulated in each strain upon *par-1* and *pos-1* dsRNA ingestion/RNAi induction. Genes were called differentially expressed if their shrunken absolute fold change was >1.5 and genome-wide adjusted *P*-value/FDR < 0.1. b) Gene set enrichment analysis results for genes upregulated on *par-1* dsRNA in each strain. GO categories that were significantly enriched (false discovery rate *Q* < 0.1) in any strain are included. GO terms are ranked and colored by median significance across strains. Related Supplementary Material: Supplementary Fig. 6 shows volcano plots for RNAi treatments for each strain. Supplementary Fig. 7 contains Venn diagrams of overlap among strains in specific differentially expressed genes. Supplementary Fig. 8 shows results from the same gene set enrichment analysis of genes downregulated under *par-1* RNAi and up- and downregulated under *pos-1* RNAi. Supplementary Table 1 gives the number of up- and downregulated genes in each strain and included in each analysis. Supplementary File 8 contains the genes differentially expressed based on strain–treatment interaction. Supplementary File 9a–9j contains the genes differentially expressed in each strain in each RNAi treatment vs control. Supplementary File 10 gives all enriched GO categories.

To assess how strains vary in overall transcriptional response to RNAi, we identified changes in gene expression across treatments (*par-1* RNAi, *pos-1* RNAi, and the negative control) that differed across the 5 strains. Specifically, for each gene in the dataset, we asked whether a model with or without a strain–treatment interaction term better explained the pattern of expression (see *Materials and methods*). Genome-wide, 842 genes (5% of those assayed) varied in RNAi response across strains (i.e. had significant strain–treatment interaction via a likelihood ratio test, genome-wide adjusted *P* < 0.1) (Supplementary File 8). We also identified, within each strain, differences in expression following *par-1* and *pos-1* RNAi relative to the control. The number of genes differentially expressed under RNAi treatment (genome-wide adjusted *P* < 0.1, fold change > 1.5) varied substantially across strains as well as between the 2 treatments (Fig. 3a, Supplementary Fig. 6, Supplementary Table 1, Supplementary File 9a–9j).

On both *par-1* and *pos-1* RNAi, the highly germline RNAi-competent strain JU1088 exhibited the most differentially expressed genes relative to the control, suggesting that this strain is the most transcriptionally responsive to RNAi (Fig. 3a, Supplementary Fig. 6). However, on *par-1* RNAi, the moderately competent strain EG4348 and the largely incompetent strains CB4856 and QX1211 showed substantially more differentially expressed genes than the competent laboratory strain N2. These results indicate that the number of genes transcriptionally responsive to exogenous RNAi is not predictive of RNAi phenotypic penetrance and that “competence” defined by endpoint phenotypes and/or artificial triggers may obscure intermediary RNAi activity or activity in alternative RNAi pathways (Chou *et al*. 2022).

Relative to *par-1*, *pos-1* RNAi induced substantially fewer differentially expressed genes in all strains but JU1088, indicating that RNAi transcriptional response is highly target-specific. Furthermore, differential expression following *par-1* RNAi was strongly skewed toward an overabundance of upregulated genes compared to downregulated genes (Fig. 3a, Supplementary Fig. 6). Of course, a transcriptional response may reflect developmental consequences of losing *par-1* or *pos-1* gene expression, at least in competent strains (Paaby *et al*. 2015; Chou *et al*. 2022); here, we cannot easily distinguish these effects from those arising from induction of the RNAi process itself. However, several lines of evidence suggest that RNAi process effects dominate. First, RNAi is a systemic phenomenon with a repertoire of many genes (Billi *et al*. 2014), while *par-1* and *pos-1* expression is largely restricted to the germline with consequential effects predominantly in the early embryo (Harris *et al*. 2020); our samples were prepared from whole worms. Second, the incompetent strains exhibited transcriptional responses genome-wide but not at the targeted genes. Finally, as described below, the transcriptional response at a gene-by-gene level was strain-specific, consistent with our growing understanding of natural variation in RNAi.

To identify transcriptional responses to RNAi that may be universal within *C. elegans*, we first checked for differentially expressed genes that were shared across strains. However, overlap among strains was sparse (Supplementary Fig. 7): no genes with differential expression to both *par-1* and *pos-1* RNAi were shared across all 5 strains, and the only gene responsive to both treatments in the competent strains (JU1088, N2, and EG4348) was *asp-14*, a predicted aspartyl protease involved in innate immunity (Harris *et al*. 2020). Such strain-specific patterns fit with our observations of RNAi variability: not only does *C. elegans* exhibit substantial natural variation in germline RNAi competence (Tijsterman *et al*. 2002; Felix 2008; Elvin *et al*. 2011; Felix *et al*. 2011; Paaby *et al*. 2015), but the genetic basis for RNAi failure appears strain-specific as well (Chou *et al*. 2022). We posit that even among competent strains, *C. elegans* varies in details of the RNAi biological response mechanism, including which genes are affected, the magnitude or functionality of their activity, and their timing. These differences are apparent in the transcriptional responses of N2 and JU1088 (Fig. 3, Supplementary Fig. 7), including the activity of W06G6.11 described above. As the RNAi response is also highly target-specific, these results portray RNAi as a phenomenon of exquisite specificity and context dependence.

However, statistical flux around significance cutoffs within strains may limit detection of gene-specific responses, and we also wished to examine the biological significance of the transcriptional responses. Therefore, we investigated whether the same general classes of genes responded to RNAi across strains by applying WormBase gene set enrichment analyses (Angeles-Albores *et al*. 2016; Harris *et al*. 2020) to the sets of genes up- and downregulated on the RNAi treatments (Supplementary File 9). Strains showed a clear pattern of enriched gene ontology (GO) categories, particularly in the largest gene set, those upregulated under *par-1* RNAi (Fig. 3b, Supplementary File 10). Specifically, GO terms associated with canonical RNAi functions such as immune defense were well represented in all strains except in the germline-incompetent strain QX1211, and genes in other categories were enriched in all strains except in N2. This pattern explains the paucity of differentially expressed genes in N2 relative to other strains following *par-1* RNAi (Fig. 3a), as those in N2 are restricted to immunity-associated ontology. These results demonstrate that reference strain N2 may not be a good representative for RNAi transcriptional response in *C. elegans* generally. Some of these patterns were also evident in genes downregulated under *par-1* RNAi and up- and downregulated under *pos-1* RNAi, though these results were less clear (Supplementary Fig. 8); this difference from *par-1* upregulated genes might reflect the more limited pool of differentially expressed genes in those categories.

In sum, transcriptional responses to RNAi differed across strains, but these responses did not clearly discriminate between RNAi-competent and RNAi-incompetent strains in the context of N2-derived GO categories: some competent strains upregulated nondefense categories while N2 did not, and incompetent strain CB4856 upregulated defense categories while incompetent strain QX1211 did not. That said, some strain-specific aspects of RNAi responses at the phenotype level may shed light on the transcriptional response enrichments. EG4348 is partially sensitive to RNAi (Felix *et al*. 2011; Paaby *et al*. 2015; Chou *et al*. 2022), and its GO term profile is similar to highly sensitive strain JU1088. While largely incompetent for germline RNAi, CB4856 does eventually exhibit strong RNAi phenotypes at late ages (Tijsterman *et al*. 2002; Felix *et al*. 2011; Paaby *et al*. 2015; Chou *et al*. 2022); its GO term profile similarity to JU1088 could be explained by the fact that this delay arises from the perturbation of a single gene, *ppw-1* (Tijsterman *et al*. 2002). Alternatively, QX1211 exhibits an apparent on/off response pattern among individual animals (Chou *et al*. 2022), and this binary penetrance of RNAi response may be insufficient to detect defense/immune gene upregulation in a bulk analysis.

### A public web resource for data exploration

We have built a user-friendly, interactive website (https://wildworm.biosci.gatech.edu/rnai/) to enable straightforward public exploration of our gene expression data across the 5 wild *C. elegans* strains and 3 RNAi conditions. For any gene in our analysis, this website (1) visualizes the RNA quantification per sample split by treatment or strain, (2) allows the user to look up differential expression results between any 2 strain–treatment groups, (3) reports if expression differs by strain in the control condition and by RNAi treatment across strains, and (4) enables initial reference bias screening by displaying DNA sequencing coverage and whether the gene overlaps a hyperdivergent haplotype. This website may be useful for exploratory analyses of genes of interest for many types of studies in the *C. elegans* community.

## Conclusion

The results of the investigations described here further expand our understanding of *C. elegans* processes beyond the reference strain N2. Our quantification of gene expression variation among wild strains demonstrates that mapping bias arising from the use of a reference genome, while a greater liability for inferences about individual genes, can be restricted to a relatively minor concern for genome-wide studies in this system. However, the strain-specific variation in RNAi transcriptomic response suggests that our understanding of RNAi processes, derived predominantly from studies in N2, incompletely represents RNAi biology in *C. elegans* as a whole. The type of dataset presented here, genome-wide expression in multiple natural genetic backgrounds over multiple conditions of interest, enables researchers to characterize how much variation exists in the experimental systems we study. Understanding the scope of natural variation informs evolutionary hypotheses about traits of interest and offers insight into otherwise inaccessible relationships among genes, their functions, and phenotypes.

## Supplementary Material

jkad112_Supplementary_Data

## Data Availability

Strains and feeding vectors are available from CeNDR or the CGC and upon request. All supplementary data files are available via Zenodo at https://doi.org/10.5281/zenodo.7406794: Supplementary File 1 contains the genes differentially expressed based on strain; Supplementary File 2 contains the “off” genes identified as potentially unexpressed in 1 strain but expressed in others; Supplementary File 3 contains raw per-gene DNA sequence coverage estimates; Supplementary File 4 contains median-normalized per-gene DNA sequence coverage estimates; Supplementary File 5 contains the list of genes flagged as low DNA coverage; Supplementary Files 6 and 7 contain summaries of missing/0-coverage genes; Supplementary File 8 contains the genes differentially expressed based on strain–treatment interaction; Supplementary File 9a–9j contains the genes differentially expressed in each strain in each RNAi treatment vs control; and Supplementary File 10 contains the results of the gene set enrichment analyses. Per-gene differential testing results and related information are available via an interactive web app at https://wildworm.biosci.gatech.edu/rnai/. Gene expression data (raw and processed) are available at GEO with the accession number GSE190803. Codes used for all analyses can be found at https://github.com/averydavisbell/wormstrainrnaiexpr. Supplemental material available at G3 online.
