# Supplementary material for: Beyond the reference: gene expression variation and transcriptional response to RNA interference in Caenorhabditis elegans
Source: G3 (Bethesda). 2023 May 23;13(8):jkad112. doi: 10.1093/g3journal/jkad112 (PMC10411595; doi:10.1093/g3journal/jkad112)
Supplement: jkad112_Supplementary_Data [file jkad112_supplementary_data.zip › jkad112_Supplementary_Data.pdf]

Supplementary Figures and Tables

Beyond the reference: gene expression variation and transcriptional response to RNAi in *C. elegans*

Avery Davis Bell, Han Ting Chou, Francisco Valencia, Annalise B. Paaby

Supplementary Figures

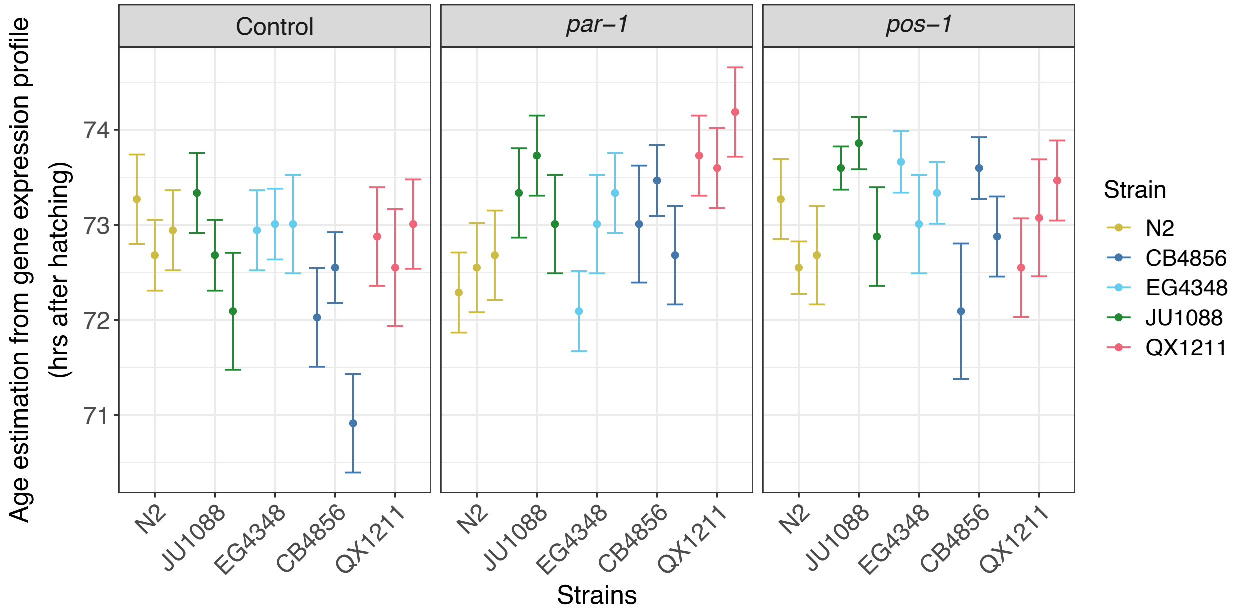

Figure S1. Estimates of sample ages from their gene expression profiles. Each expression profile was compared to time course gene expression from N2 worms using RAPToR (Bulteau and Francesconi 2022). Each point represents a biological replicate; error bars are 95% confidence intervals generated by RAPToR's bootstrapping.

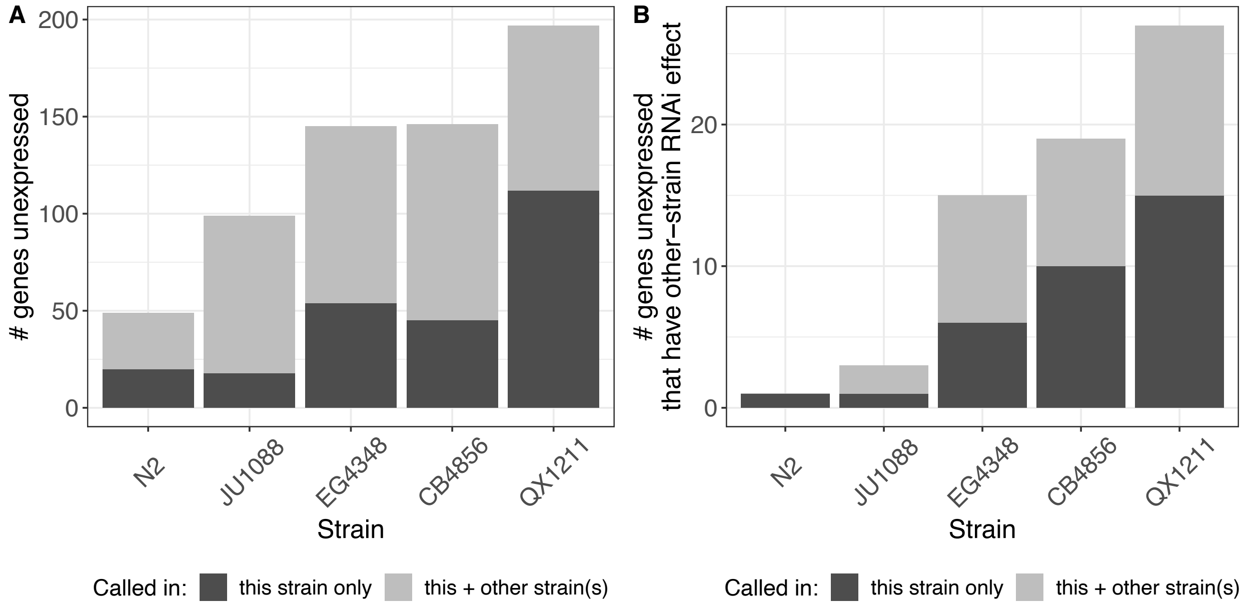

Figure S2. 'Off' genes, which are expressed in at least one strain but show no expression in one or more others. **A)** All 'off' genes per strain, either unique or shared across strains (n = 411 total; genes may be present for multiple strains). **B)** The subset of 'off' genes that exhibit differential expression on RNAi to *par-1* or *pos-1* in other strains, which are potential candidates for RNAi functional divergence (n = 47 total; genes may be present for multiple strains).
File S2 contains identity and details for each of these 'off' genes.

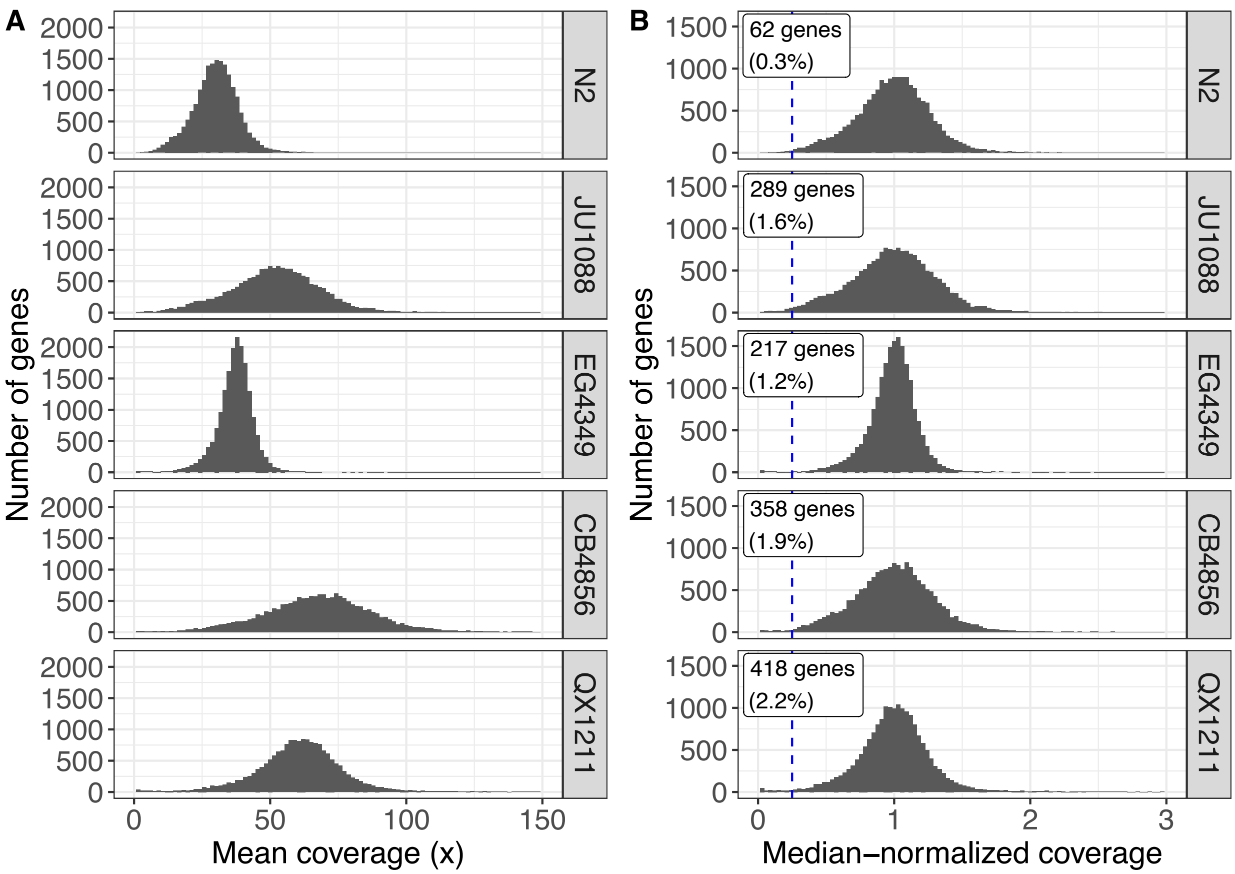

Figure S3. DNA sequence coverage across 18,589 genes included in expression analyses. Aligned DNA sequence data was obtained from CeNDR (release 20210121) (Cook et al. 2017). **A)** Mean coverage (mean number of reads covering each base) over merged non-overlapping exonic regions of genes in the five strains in this study. CeNDR assessed DNA coverage in EG4349, the genetically identical isotype to EG4348. The x-axis is truncated at 150x coverage for visual clarity, excluding 179 genes across all strains combined. **B)** Median-normalized coverage for the same genes as in **(A).** Genes with less than 25% median coverage are considered low DNA coverage in this study; this boundary is demarcated with the blue dashed line and the number and proportion of genes this set comprises is noted on the plots. The x-axis is truncated at 3x median coverage for visual clarity, excluding 227 genes across all strains combined.

Files S3 and S4 contain the source data. File S5 provides the list of genes identified as low coverage.

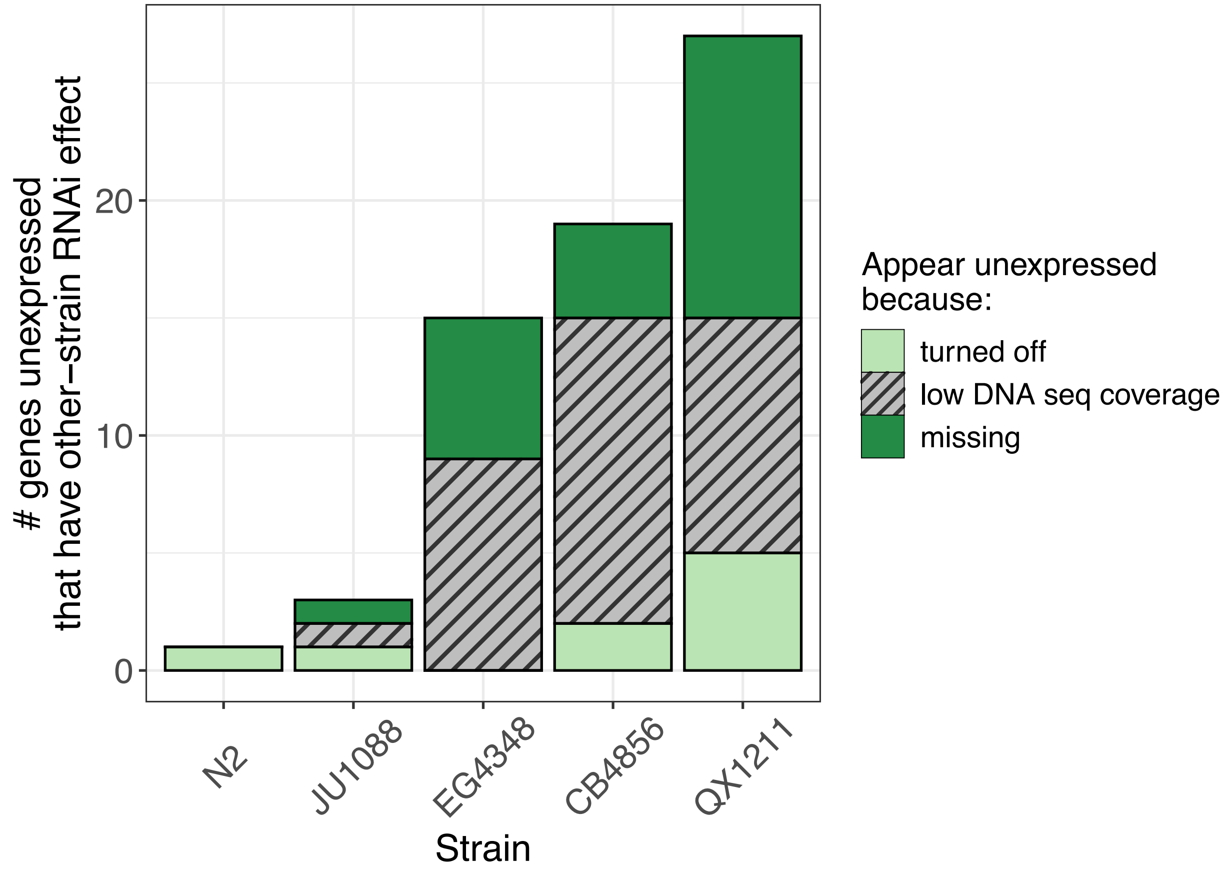

Figure S4. ‘Off’ genes that were unexpressed in one or more strains but differentially expressed with respect to *par-1* or *pos-1* RNAi in another strain, potential candidates for RNAi functional divergence. DNA sequence coverage information is denoted with color and shading. Missing genes were those with zero DNA sequence coverage; low DNA sequence coverage genes had greater than zero but less than 25% median gene’s coverage; genes classified as truly turned off had greater than 25% median gene’s DNA sequence coverage. (DNA coverage was assessed in strain EG4349, isotype to EG4348).

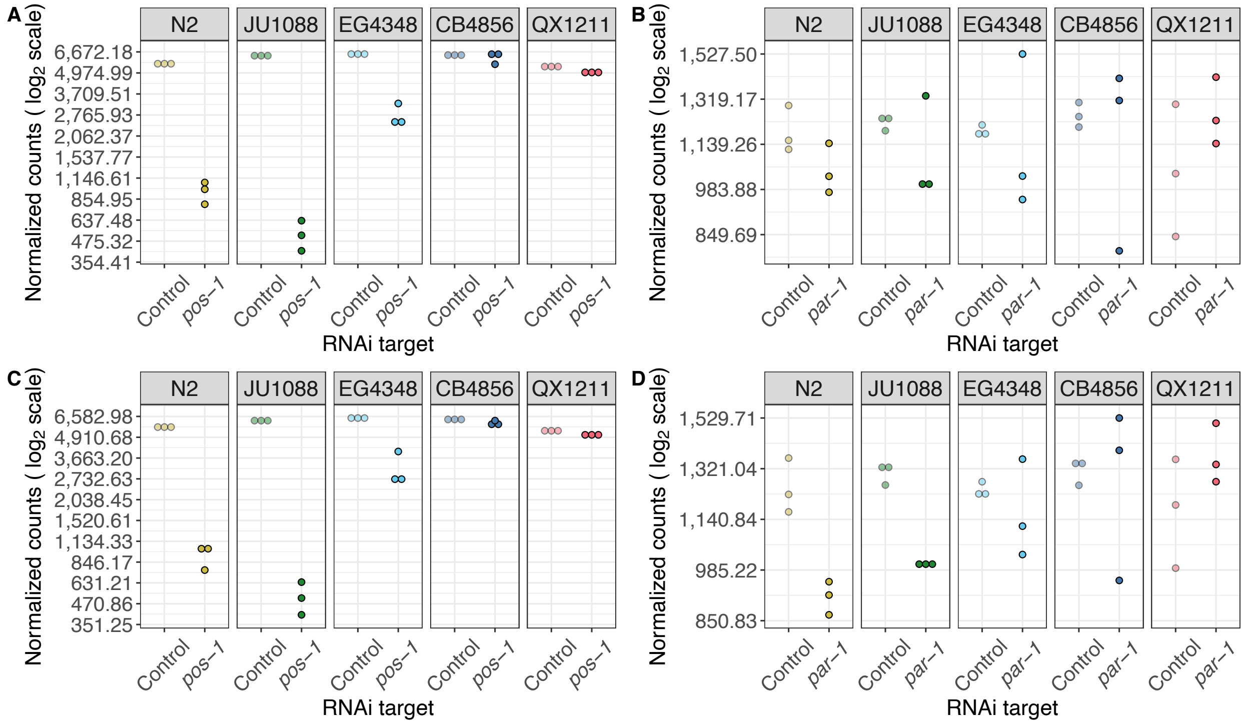

Figure S5*.* RNA-seq estimates suggest RNAi targets are knocked down commensurate with each strain's RNAi capacity. **(A and B)** Quantification estimates from pseudoalignment to strain-specific transcriptomes, normalized to library size and gene length, as used for all analyses in this study. **A)** Quantification estimates for *pos-1* in control and exposure to *pos-1* dsRNA; response is significantly different across strains (the strain:treatment interaction is significant, genome-wide adjusted p = 4 x 10^{-254}). **B)** Quantification estimates for *par-1* in control and exposure to *par-1* dsRNA (the strain:treatment interaction is not significant, genome-wide adjusted p = 0.92). **(C and D)** Detection of target knockdown is not dependent on RNAi strategy: panels show *pos-1* and *par-1* quantification estimates as in (**A and B**), respectively, but with alternative expression estimates derived from RNA sequence data uniquely mapping to one genomic location when containing the reference or non-reference allele (see methods).

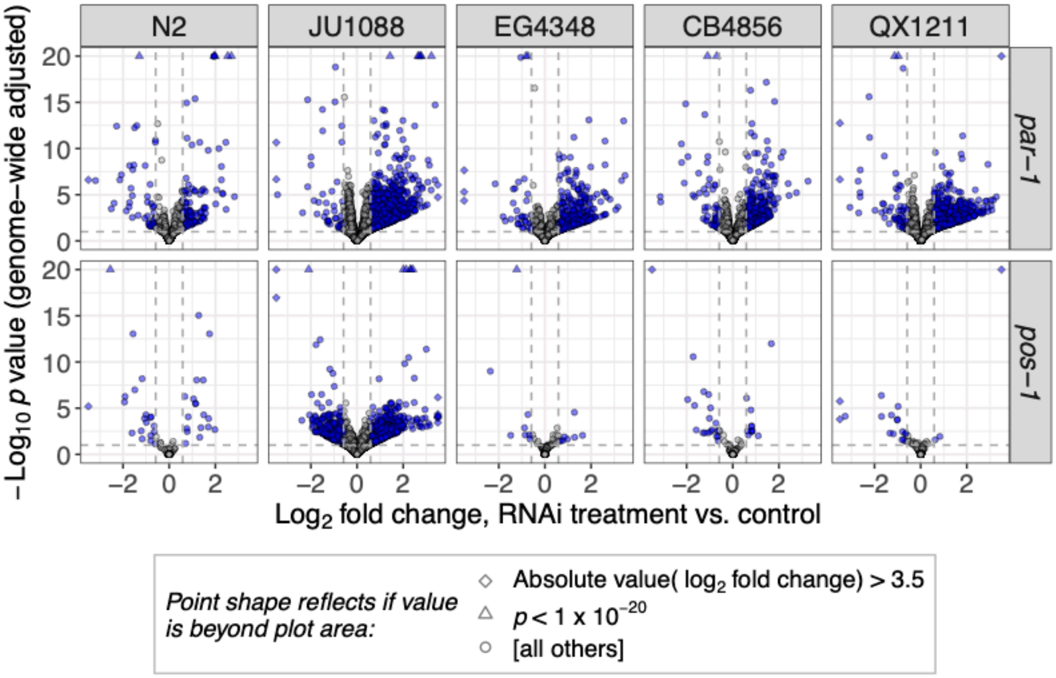

Figure S6. Volcano plots show genome-wide effects of RNAi treatments (against *par-1*, top, and *pos-1*, bottom) in each of the five strains. All genes with differential expression estimates are plotted; blue points denote genes with significant differential expression (genome-wide adjusted $p < 0.1$ and corrected [see methods] absolute value (fold change) > 1.5; these thresholds are annotated on the plot with gray dashed lines). For visual clarity, the y-axis is truncated at $p = 10^{-20}$ and the x-axis is truncated at absolute log_2 fold change = 3.5; genes with values exceeding these thresholds are included on the plots and are represented by unique point shapes as noted in the plot legend.

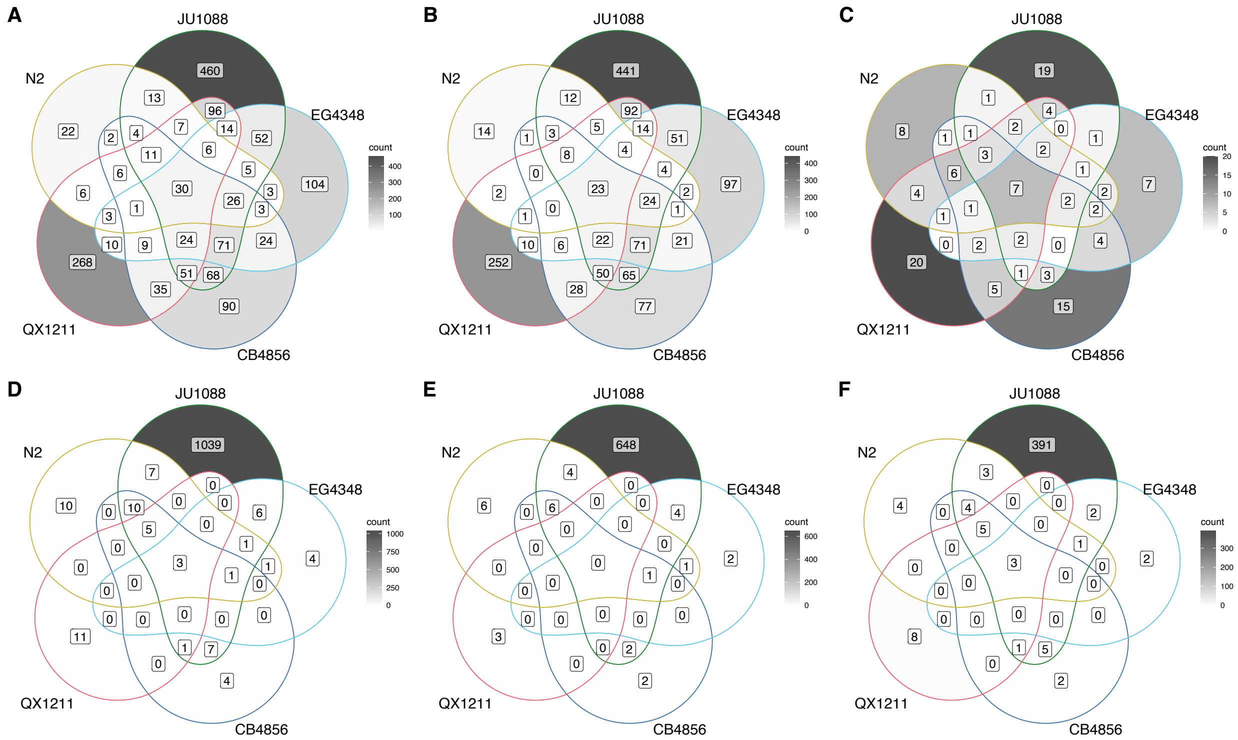

Figure S7. Limited overlap of genes called as differentially expressed in RNAi conditions vs. control across strains; shading scales with number of genes separately within each panel (see color bar legends). (**A-C)** Under *par-1* RNAi, genes differentially expressed in either direction **(A)**, upregulated **(B),** or downregulated **(C)**. **(D-F)** Under *pos-1* RNAi, genes differentially expressed in either direction **(D)**, upregulated **(E),** or downregulated **(F)**. Genes were called differentially expressed and included if their shrunken absolute fold change was > 1.5 and genome-wide adjusted p-value/FDR < 0.1 between RNAi and control within-strain.

Files S9a-j contain gene IDs and details. Figure 3A and Table S1 show the overall number of up- and down-regulated genes in each strain.

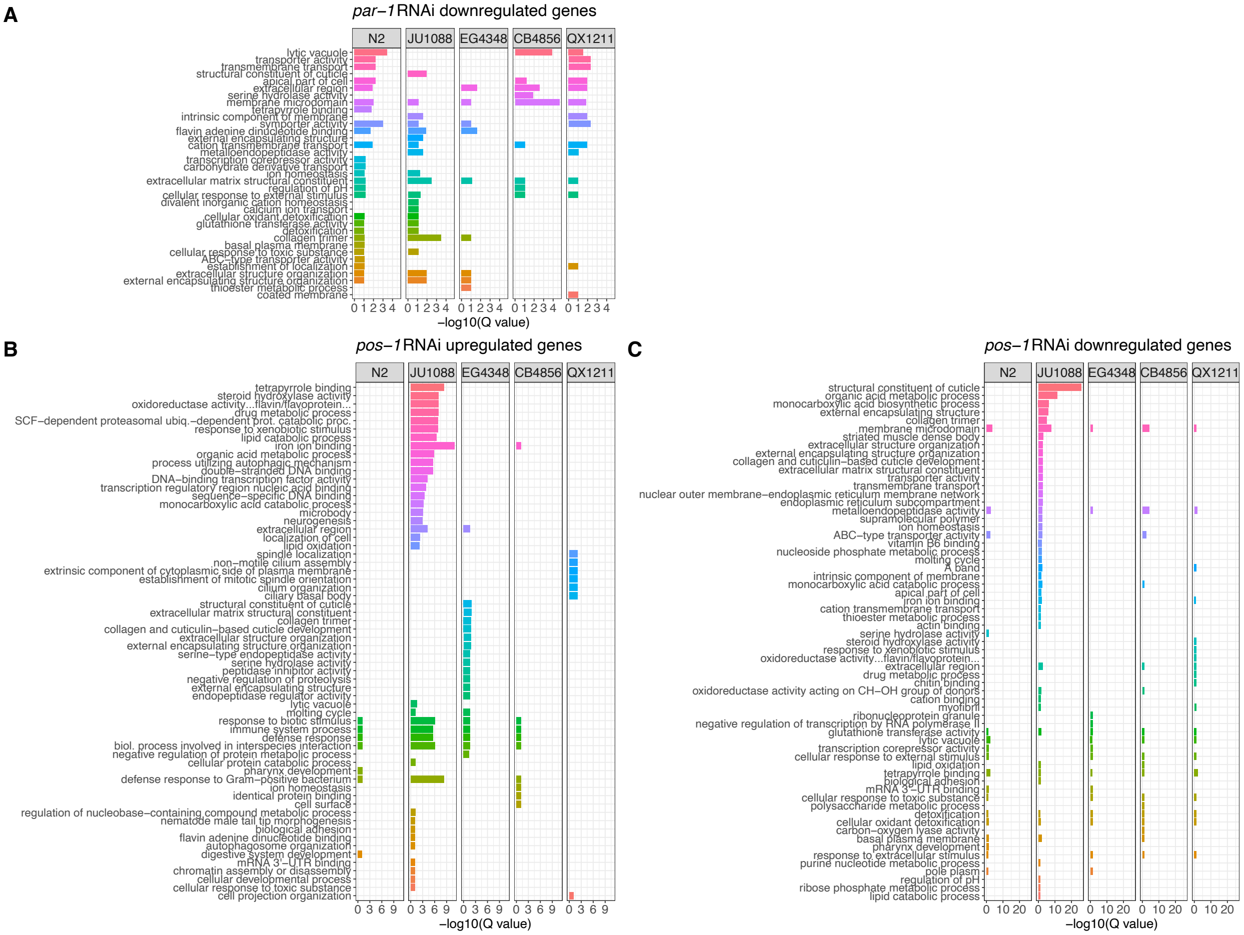

Figure S8. Gene set enrichment analysis results for genes **(A)** downregulated on *par-1* dsRNA in each strain, (**B**) upregulated on *pos-1* dsRNA, and (**C**) downregulated on *par-1* dsRNA. Only gene ontology (GO) categories significantly enriched (FDR Q < 0.1) in upregulated genes in any strain are included. GO terms are ranked and colored by median significance across strains. *Table S1 provides the number of genes included for each analysis. File S10 gives all enriched GO categories. Main Fig 3B displays the same analysis of genes upregulated under* par-1 *RNAi.*

Supplementary Tables

Table S1. The number of genes differentially expressed in each RNAi treatment in each strain, relative to the control condition, as well as the number included in the gene set enrichment analysis (GSEA).

RNAi Treatment	Strain	Up- or down-regulated vs. control-treated samples	N genes significantly up- or downregulated*	N genes included in GSEA testing	N genes excluded from GSEA testing**
par-1	CB4856	Down	55	35	20
		Up	400	282	118
	EG4348	Down	34	22	12
		Up	351	222	129
	JU1088	Down	49	29	20
		Up	909	569	340
	N2	Down	44	31	13
		Up	104	62	42
	QX1211	Down	60	46	14
		Up	517	380	137
pos-1	CB4856	Down	20	17	3
		Up	11	5	6
	EG4348	Down	8	7	1
		Up	8	6	2
	JU1088	Down	415	315	100
		Up	665	394	271
	N2	Down	20	16	4
		Up	18	7	11
	QX1211	Down	17	15	2
		Up	3	2	1

*, up or downregulation means shrunken fold change > 1.5 or < 1.5, respectively, and genome-wide adjusted p-value < 0.1
**, genes excluded by WormBase due to lack of association with any WormBase gene ontology category
